# Muscle degeneration in aging *Drosophila* flies: the role of mechanical stress

**DOI:** 10.1186/s13395-024-00352-4

**Published:** 2024-08-20

**Authors:** Maria Chechenova, Lilla McLendon, Bracey Dallas, Hannah Stratton, Kaveh Kiani, Erik Gerberich, Alesia Alekseyenko, Natasya Tamba, SooBin An, Lizzet Castillo, Emily Czajkowski, Christina Talley, Austin Brown, Anton L. Bryantsev

**Affiliations:** 1 Department of Molecular and Cellular Biology, Kennesaw State University, 105 Marietta Dr., NW, Room 4004, MD 1201, Kennesaw, GA 30144 USA; 2 Department of Biology, University of New Mexico, Albuquerque, NM USA; 3 Department of Mathematics, Kennesaw State University, Kennesaw, GA USA; 4 Present Affiliation: Department of Molecular Biosciences, Northwestern University, Evanston, IL USA; 5 Present Affiliation: MNG Laboratories, A LabCorp Company, Atlanta, GA USA

**Keywords:** Sarcopenia, *Drosophila*, Muscle, Aging, Muscle fiber, Degeneration, Necrosis, Mechanical stress

## Abstract

Muscle wasting is a universal hallmark of aging which is displayed by a wide range of organisms, although the causes and mechanisms of this phenomenon are not fully understood. We used *Drosophila* to characterize the phenomenon of spontaneous muscle fiber degeneration (SMFD) during aging. We found that SMFD occurs across diverse types of somatic muscles, progresses with chronological age, and positively correlates with functional muscle decline. Data from vital dyes and morphological markers imply that degenerative fibers most likely die by necrosis. Mechanistically, SMFD is driven by the damage resulting from muscle contractions, and the nervous system may play a significant role in this process. Our quantitative model of SMFD assessment can be useful in identifying and validating novel genetic factors that influence aging-related muscle wasting.

## Introduction

Complications arising from degeneration of skeletal muscles compromise physical well-being, quality of life, and ultimately can lead to death [1–3]. Virtually all animals, from worms to mammals, experience some degree of muscle loss during their lifetime [4–7]. In humans, gradual but progressive decline of muscle mass is associated sarcopenia, a pathological condition affecting a broad population of older individuals world-wide [8, 9].

Changes in gross muscle size and mass are reflective of underlying microscopic alterations occurring at the level of individual cellular units known as muscle fibers. While atrophic changes affecting the size of individual fibers are often reversible, dystrophic degenerative changes present a lasting damage due to the physical elimination of muscle fibers. While mammalian muscle demonstrates remarkable regenerative potential in response to acute injury [10], this capacity diminishes with chronic injury or advancing age [11, 12]. Consequently, losses in muscle fibers may gradually accumulate over the lifetime to reach significant changes, as it has been shown for leg muscles in aging humans and mice in whole-muscle quantification assays [6, 13, 14]. However, the precise nature and underlying causes of muscle fiber decline remain incompletely understood.

*Drosophila* somatic muscles closely resemble mammalian skeletal muscles and consist of striated, multi-nucleated fibers characterized by highly organized arrays of contracting myofibrils, T-tubules, and neuromuscular junctions. The contractile apparatus in flies also exhibits a high degree of conservation, and can be visualized using phalloidin, a natural compound that selectively binds to filamentous, polymerized actin (F-actin), and equally well labels muscles in flies and mammals. Moreover, adult fly muscles demonstrate a remarkable degree of specialization and diversity. A classic example of two strikingly different muscle types in flies is the Indirect Flight Muscles (IFMs) that are responsible for powering flight and exhibit high-frequency oscillations, and the tergal depressor of the trochanter (TDT), or jump muscle, that contracts infrequently and is required for occasional escape response and flight initiation [15]. IFMs and TDTs differ substantially by their mitochondrial content and muscle protein isoforms, resembling the differences displayed by slow- and fast-twitch fibers of mammalian muscle [16–18]. Overall, *Drosophila* muscles exhibit strong parallels with mammalian muscle across multiple levels of organization with one substantial exception – they do not undergo active repair.

*Drosophila* is employed to study various aspects of muscle aging, encompassing protein aggregation, mitochondrial damage, oxidative stress, etc. (reviewed in [19]). Numerous electron microscopy studies in the last century have investigated ultrastructural changes in aging muscles of flies [20–22], revealing usually mild alterations such as glycogen granule loss and disarrayed mitochondrial cristae. However, in select cases or in extremely aged flies, degenerative changes were shown to escalate dramatically, leading to complete contractile apparatus disintegration. These early studies have underscored the stochastic nature of muscle degeneration in adult flies and confirmed that damaged fibers have no active repair. Nonetheless, a limitation of these studies was the disproportionate focus on IFMs and a lack of systematic observations involving other somatic muscles.

In this study, we demonstrate that diverse adult muscles in *Drosophila* undergo spontaneous muscle fiber degeneration (SMFD), a stochastic process occurring in individual muscle fibers and involving compromised cellular membranes. Through quantitative analysis, we established a correlation between SMFD and both chronological and functional aging in flies. Our findings provide mechanistic insights into the origins of SMFD and suggest the involvement of the nervous system in this process. Notably, we discovered that SMFD rates are influenced by genotype, paving the way for future investigations to identify genetic determinants implicated in development of sarcopenia and muscle fiber loss.

## Materials and methods

### Fly stocks and husbandry

Flies were cultured on Jazz mix food (Fisher) on a 12-h light/dark cycle. For aging studies, 1–2 day old adults were placed in standard plastic vials (Genesee Scientific) at a density of 35 flies/vial, and kept at 29 °C while changing the vials twice a week. This temperature was higher than the optimal temperature for *Drosophila* culturing (*i.e.*, 25 °C), but it significantly shortened the turnaround time for aging trials. In a preliminary study, we observed the same effects on muscle aging in flies aged at 29 °C and 25 °C. For aging females, three male mating partners were additionally added per vial. Most fly lines were supplied by the Bloomington *Drosophila* Stock Center (BDSC); Table 1 lists details of the fly lines used in this study. Genetic crosses were set up at 25 °C and newly eclosed adults were transferred to 29 °C for aging. A control cross for RNAi knockdown experiments was set between a driver line and *attP2* flies for the best genetic background matching (see Table 1). The mechanical stimulation applied in the experiments involving bang-sensitive flies was described in Horne et al. as “vortex testing” [23]. In brief, a standard culture vial containing flies was shaken for 10 s using a lab vortexer set to maximum speed. Such treatment was repeated daily for the entire duration of aging trial (4 weeks).

**Table 1:** Genetic fly lines used in this study

Short ID	Source	Genetic background	Notes
*w*^*1118*^	Gift from Dr. Young Kwon, Harvard Medical School	*w*[1118]	Used as a reference line in aging experiments
*y w*	Gift from Dr. Richard Cripps, University of Sand Diego	*y*[*] *w*[*]	
DGRP-21	BDSC 28122		Inbred wild-type strain
DGRP-31	BDSC 55014		Inbred wild-type strain
DGRP-41	BDSC 28126		Inbred wild-type strain
DGRP-45	BDSC 28128		Inbred wild-type strain
DGRP-57	BDSC 29652		Inbred wild-type strain
DGRP-59	BDSC 28129		Inbred wild-type strain
DGRP-75	BDSC 28132		Inbred wild-type strain
DGRP-85	BDSC 28274		Inbred wild-type strain
DGRP-88	BDSC 28135		Inbred wild-type strain
DGRP-91	BDSC 28136		Inbred wild-type strain
DGRP-93	BDSC 28137		Inbred wild-type strain
DGRP-101	BDSC 28138		Inbred wild-type strain
DGRP-105	BDSC 28139		Inbred wild-type strain
DGRP-109	BDSC 28140		Inbred wild-type strain
DGRP-136	BDSC 28142		Inbred wild-type strain
DGRP-142	BDSC 28144		Inbred wild-type strain
DGRP-149	BDSC 28145		Inbred wild-type strain
DGRP-153	BDSC 28146		Inbred wild-type strain
DGRP-158	BDSC 28147		Inbred wild-type strain
DGRP-161	BDSC 28148		Inbred wild-type strain
DGRP-177	BDSC 28150		Inbred wild-type strain
DGRP-208	BDSC 25174		Inbred wild-type strain
DGRP-287	BDSC 28165		Inbred wild-type strain
DGRP-303	BDSC 25176		Inbred wild-type strain
DGRP-306	BDSC 37525		Inbred wild-type strain
DGRP-307	BDSC 25179		Inbred wild-type strain
DGRP-309	BDSC 28166		Inbred wild-type strain
DGRP-310	BDSC 28276		Inbred wild-type strain
DGRP-313	BDSC 25180		Inbred wild-type strain
DGRP-318	BDSC 28168		Inbred wild-type strain
DGRP-319	BDSC 55018		Inbred wild-type strain
DGRP-320	BDSC 29654		Inbred wild-type strain
DGRP-324	BDSC 25182		Inbred wild-type strain
DGRP-332	BDSC 28171		Inbred wild-type strain
DGRP-336	BDSC 28172		Inbred wild-type strain
DGRP-338	BDSC 28173		Inbred wild-type strain
DGRP-340	BDSC 28174		Inbred wild-type strain
DGRP-348	BDSC 55019		Inbred wild-type strain
DGRP-350	BDSC 28176		Inbred wild-type strain
DGRP-360	BDSC 25186		Inbred wild-type strain
DGRP-382	BDSC 28189		Inbred wild-type strain
DGRP-409	BDSC 28278		Inbred wild-type strain
DGRP-820	BDSC 25208		Inbred wild-type strain
DGRP-852	BDSC 25209		Inbred wild-type strain
79B6	[24]	w[1118] P(w[+ mC] = Act79B-Gal4)6	TDT-specific driver for the Gal4/UAS system, employed in TDT-specific knockdowns
*elav-Gal4*	BDSC 458	P([+ mW.hs] = GawB)elav[C155]	Neuron-specific driver for the Gal4/UAS system, employed in KD^NEU^ flies
*Mef2-Gal4*	Gift from Dr. Aaron Johnson, Washington University in St. Louis	w[*];P(w[+ m*] = Mef2-Gal4)3	Muscle-specific driver for the Gal4/UAS system, used in KD^MUS^ and mito-GFP flies
*Ca-α1D KD*	BDSC 33413	y[1] v[1]; P(y[+ t7.7] v[+ t1.8] = TRiP.HMS00294)attP2	Induces Ca*-α1D* RNAi knockdown
*jus KD*	BDSC 28764	y[1] v[1]; P(y[+ t7.7] v[+ t1.8] = TRiP.JF03192)attP2	Induces *jus* RNAi knockdown
*attP2*	BDSC 36303	y[1] v[1]; P(y[+ t7.7] = CaryP)attP2	Control for RNAi knockdown crosses
*mito-GFP*	BDSC 8442	w[1118]; P(w[+ mC] = UAS-mito-HA-GFP.AP)2/CyO	Expresses mitochondrial fluorescent marker [25]

### Functional tests

For jumping testing, flies with clipped wings were allowed to freely walk on a sheet of plain paper. Individual flies were stimulated to jump by gently touching their abdomen with a paintbrush; the takeoff and landing spots were marked by pencil and were used to calculate the jumped distance. The average distance obtained from three jumping attempts was recorded for each fly. For flight testing, flies were released from a vial held in the center of a plastic box with graded landing zones: upward (U), horizontal (H), down (D), or null (N) that was described previously [26]. Each fly’s performance was scored based on the landing location, and the flight index was calculated according to the formula: (6x[U flies] + 4x[H flies] + 2x[D flies] + 0x[N flies])/[all tested flies] [27].

### Cryosectioning and immunofluorescence

We followed general guidelines of sample preparation, as previously described [28]. Cryosections of flies were produced in the horizontal plane, at 7–10 µm thickness, and air-dried on standard microscopy slides (SuperFrost Plus, Fisher) We used the following mouse monoclonal antibodies from the Developmental Studies Hybridoma Bank as primary antibodies: anti-Dlg (clone 4F3), anti-α integrin (clone DK.1A4), and anti-β-integrin (clone CF.6G11). Incubation with primary antibodies (diluted 1:50) was done overnight in a staining solution (Phosphate Buffered Saline (PBS) supplemented with 0.1% Triton X-100 and 1% Bovine Serum Albumin (BSA)), in a humid chamber at room temperature. For anti-integrin staining, the two monoclonal antibodies were combined and applied simultaneously. For visualization, secondary Cy3-labeled goat anti-mouse antibody (115–167-003, Jackson ImmunoResearch) was incubated in the staining solution (without BSA) for 1 h at room temperature. For muscle and nuclear counterstaining, we used phalloidin conjugated with iFluor 488 (ab176753, Abcam) and DAPI (Sigma), respectively.

### Vital dye injections and analysis

Anesthetized flies were covered by the Optimal Cutting Temperature (OCT) medium used for cryosectioning (TissueTek) and placed on a metal spatula with the ventral side facing up. Trypan blue (2.5% solution) was injected into the abdomen via a glass capillary needle using micromanipulators and a pneumatic injector (Narishige). The process of dye filling was monitored under a dissecting microscope. Flies that acquired blue staining through the thorax and head were immediately flash-frozen in liquid nitrogen. Air-dried 10-um cryosections of the injected flies were imaged using bright filed microscopy and their position was recorded using microscope stage micrometers. The slides were then fixed and processed for staining with phalloidin and DAPI as described above; the blue dye was washed away during the process. The same sections were then identified by recorded stage coordinates and re-imaged using fluorescence microscopy.

### Histochemistry

We essentially followed previously published protocols [17, 29]. In brief, microscopy slides containing fresh, 15-µm cryosections were incubated at room temperature in the staining solution (50 mM Tris pH7.4, 1 mg/mL nitro blue tetrazolium chloride, 5 mM MgCl2, 50 mM sodium succinate, 10 mM sodium azide). Sections from young and old flies were stained in parallel. Staining progress was monitored under a dissecting microscope; reactions were terminated by moving the slides into a fixing solution (4% formaldehyde in PBS) for 1o min.

### Microscopy and image acquisition

AxioImager 2 (Zeiss) equipped with 20X/0.8 NA objective and color and monochrome CCD cameras (Axiocam HR and Axiocam MR, Zeiss) was used for routine examination of slides. Select samples were imaged with the laser confocal microscope LSM 700 (Zeiss). Image acquisition was done via the Zen software (Zeiss). Image cropping and image intensity adjustments for figures were done in Photoshop (Adobe).

### Nuclear content quantification

Images of nuclei were encircled and their mean pixel intensity in the blue channel was determined by the Zen software. Signal intensity from degenerative nuclei was expressed as percentage of the intensity from neighboring nuclei located in intact fibers of the same TDT muscle. A minimum of 10 degenerative fibers from different flies were analyzed per condition.

### Quantification of muscle fiber loss

Typically, 15 flies (*i.e.,* 30 TDTs or subalar muscles) were analyzed per sample. Every muscle was imaged 2 or 3 times from tissue sections obtained at various depths within the thorax. Two researchers independently analyzed each image to count intact (live) fibers and to call a degeneration score. Special care was taken to recognize and exclude mechanical artifacts, such as tissue tears, from the analysis. If two images of the same muscle showed different levels of degeneration, the more severe damage was recorded. To calculate the mean of aging-related changes in live TDT fibers (Δ$\overline{\text{N} }$), the mean of fiber counts obtained from the young flies ($\overline{\text{N} }$[young]) was subtracted from individual fiber counts (N_1_[old]… N_n_[old]) obtained from old TDTs (n) and then the average of individual differences was calculated, according to the formula: Δ$\overline{\text{N} }$ = ((N_1_[old]-$\overline{\text{N} }$[young]) + … + (N_n_[old]-$\overline{\text{N} }$[young]))/n.

For the degeneration scoring system, muscles with all fibers intact received a score of “0”, muscles with a single degenerate or dead fiber received a score of “1”, muscles with multiple degenerate fibers (typically 2–4) received a score of “2”, and muscles that were either no longer holding together as a whole or where the damage affected more than 50% of the cross-sectioned area received a score of “3”. The distribution of scores within a sample was depicted in graphs by circles, with the area of each circle representing the proportion of muscles that received each score. The sum of all circle areas equaled 100%, representing all muscles analyzed in the sample. To calculate the overall degeneration score for a particular genetic line, scores obtained from all assessed TDT muscles in that line were averaged.

### Statistical analysis and calculations

Box plots were created with the online tool described previously [30]. Samples with normal distribution were compared using the Student’s *t-*test. Jump test results were assessed by the Kruskal–Wallis test followed by the Dunn’s post-hoc analysis. Data from the climbing tests and muscle degeneration scores were compared using the Fisher’s Exact Test. Differences were deemed statistically non-significant with *p* > *0.05*. Statistically significant differences were denoted by * (for *p* < *0.05*), ** (for *p* < *0.01*), and *** (for *p* < 0.001).

## Results

### Muscles of the dorsal thorax

**Fig. 1:**
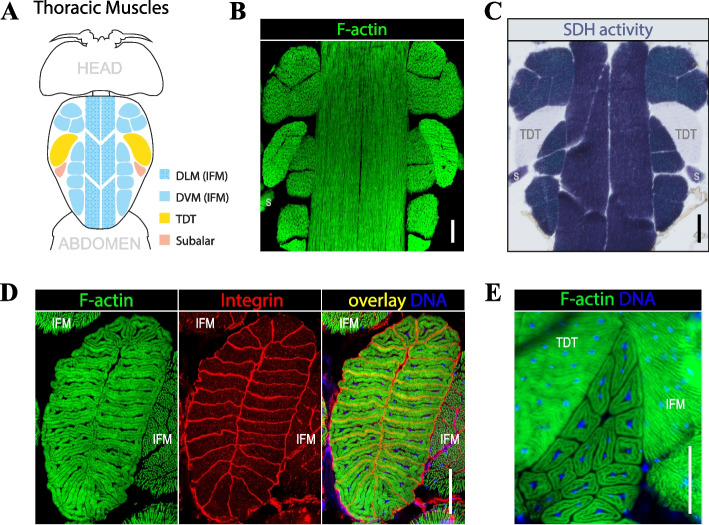
Thoracic muscles in *Drosophila*. **A** Organization of muscles in the dorsal part of the thorax. Dorsal longitudinal (DLM) and Dorsoventral muscles (DVM), belonging to the Indirect Flight Muscles (IFM), as well as the jump muscles (TDT) and subalar muscles are color coded. **B** Thoracic muscles revealed by F-actin staining via fluorescently labeled phalloidin. **C** Thoracic muscles histochemically stained for succinyl dehydrogenase (SDH) activity. TDT muscles naturally have low SDH activity (lighter blue), while IFMs and subalar (s) muscles display stronger SDH activity (darker blue). **D**, **E** The multi-fiber composition of tubular muscles revealed by F-actin (green), integrin (red), and DNA (blue) staining. Note the differences in fiber morphology of the jump (**D**) and subalar (**E**) muscles. Scale bars: 50 µm (all panels)

Longitudinal horizontal sections through the dorsal portion of fly thorax reveal a stereotypic muscle pattern consisting of large IFM fibers (represented by two groups, DLMs and DVMs), a pair of TDTs, and adjacent subalar muscles (Fig. 1A). All these muscles can be readily detected by uniform F-actin staining using fluorescently labelled phalloidin (Fig. 1B). Each muscle group, however, has a unique set of characteristics. IFMs and subalar muscles demonstrate strong and moderate SDH activity, respectively, while TDTs have very low SDH activity (Fig. 1C). From another hand, TDTs and subalar muscles share similar morphology featuring centrally located myonuclei surrounded by tightly linked phalloidin-positive myofibrils, whereas IMFs have a distinct appearance with myonuclei scattered between loosely arranged myofibrils (Fig. 1D, E).

A common feature of all these muscles is that they are composed of multiple individual fibers, which can be distinguished by immunostaining of the surface receptors integrins. TDTs contain numerous fibers, each consisting of 20–35 tightly packed fibers (Fig. 1D). A sigle subalar muscle typically comprises 10–15 fibers that are readily distinguishable by F-acting staining alone (Fig. 1E). IFMs have larger but fewer fibers: DVMs are represented by 14 loosely distributed fibers and DLMs have 12 fibers, although not all of them can be simultaneously visualized from horizontal sections due to their horizontal orientation in the thorax (Fig. 1A, B, C).

Thus, the dorsal part of thorax is a convenient location in the fly body where several diverse and multi-fiber muscles can be simultaneously assessed for quantitative analysis.

### Detection of SMFD in flies

**Fig. 2:**
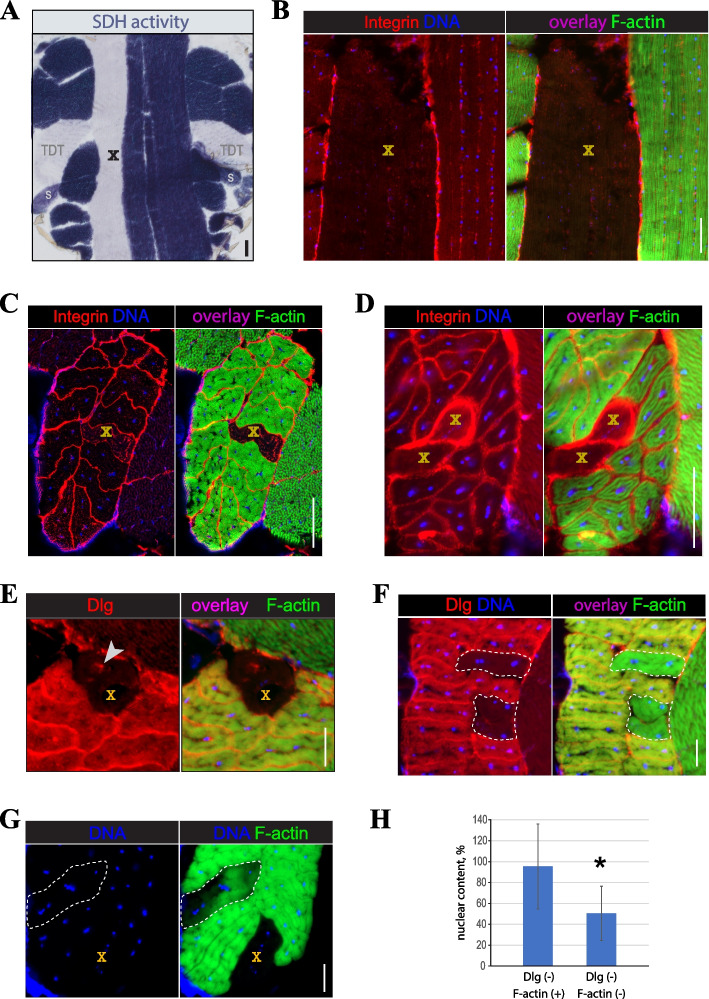
Markers of spontaneous muscle degeneration in flies. **A** The loss of SDH staining in a degenerative DLM fiber. **B**-**D** The loss of F-actin (green) staining and reduced DNA (blue) staining in degenerative muscle fibers of IFM (**B**), TDT (**C**), and subalar (**D**) muscles. “TDT” and “s” mark positions of TDT and subalar muscles, respectively. Integrin staining (red) shows individual muscle fibers. **E**–**G** Dynamics of T-tubule (Dlg, red), F-actin (green), and DNA (blue) staining in degenerating TDT muscles. Note that loss of Dlg staining precedes disappearance of F-actin staining. Arrowhead indicates accumulation of Dlg in an internal vacuole; “X” marks degenerative muscle fibers; dashed line outlines degenerative fibers with no or incomplete loss of F-actin staining. **H** Changes in the DNA content of the myonuclei from degenerative fibers. Average intensities of DNA staining from micrographs of degenerating (Dlg-, F-actin +) TDT muscles was normalized by the signal from neighboring intact (Dlg + , F-actin +) muscle fibers. Multiple fibers from 10 flies have been analyzed in each category. Statistical significance by the Student’s *t*-test: (*) *p* < 0.05. Scale bars: 50 µm (**A**-**D**), 20 µm (**E**–**G**)

Upon systematic review of thoracic sections, occasional degenerative fibers can be routinely identified in all thoracic muscles. Degenerative fibers lose SDH activity, F-actin staining, and nuclear content (Fig. 2A, B). Fiber degeneration occurs fiber-autonomously and usually does not affect adjacent fibers within the same muscle (Fig. 2C). Nevertheless, multi-degeneration events involving two or more fibers within the same muscle can also occur (Fig. 2D).

By analyzing TDT images depicting T-tubules, F-actin, and nuclear DNA, we determined the sequence of events that take place in degenerating fibers. T-tubules form a dense network in muscle fibers to propagate the action potential and initiate muscle contraction [31]. Live TDT fibers are strongly positive for Discs large (Dlg), a molecular marker of T-tubules [32], but in degenerated fibers Dlg staining was lost along with F-actin and nuclear staining (Fig. 2E). In some cases, the loss of Dlg staining preceded the loss of F-actin staining, but not vice versa (Fig. 2F). Nuclear DNA fluorescence undergoes a visible reduction in degenerating fibers, although at a slower rate and magnitude than other markers (Fig. 2G). In Dlg-negative but F-actin-positive fibers, DNA fluorescence is not changed significantly and remains close to control values, but in Dlg, F-actin double-negative fibers, DNA fluorescence intensity is declined significantly (Fig. 2G, H). Notably, despite the reduced DNA levels, myonuclei in degenerative fibers do not undergo visible fragmentation.

Collectively, these observations establish a sequence of degenerative events affecting *Drosophila* adult muscles, in which the T-tubule network is lost first, followed by a loss of F-acting staining and, later, by a loss of nuclear content.

### SMFD is driven by necrosis

**Fig. 3:**
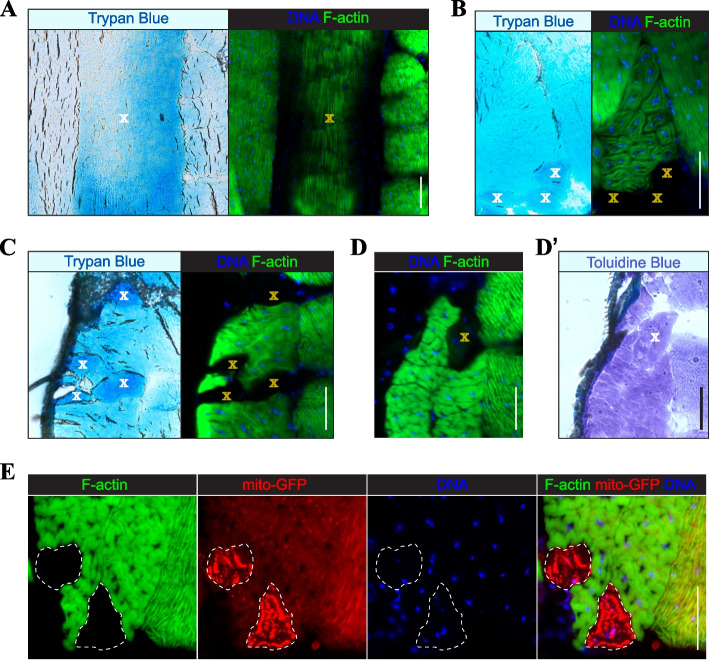
Degenerative fibers in *Drosophila* muscles show signs of necrosis. **A**-**C** Accumulation of injected vital dye trypan blue in degenerative fibers of IFM (**A**), subalar (**B**), and TDT (**C**) muscles. D-D’: A degenerative TDT fiber that lacks F-actin and DNA staining (**D**) does not show enhanced accumulation of apoptosis-selective dye toluidine blue in a serial section (D’). “X” marks degenerative fibers as determined by reduced or lacking staining for F-actin (green) and DNA (blue). **E** Mitochondrial swelling in two degenerative TDT muscle fibers (outlined) expressing mito-GFP (red). Scale bars: 50 µm (all panels)

Muscle death can be inflicted by a damage involving the outer membrane (*i.e.,* sarcolemma), leading to necrosis, or by activation of intrinsic program, resulting in apoptosis [33]. To better understand, which death mechanism underlies SMFD in flies, we conducted experiments with selective dye accumulation. Trypan blue and its close derivate Evans blue are vital dyes that accumulate in cells with damaged plasma membrane [34]. When trypan blue was injected into live flies, it preferentially accumulated in the muscle fibers that showed signs of degeneration. Muscle fibers from IFMs, subalar, and TDT muscles that lacked or had significantly reduced F-actin and nuclear staining readily accumulated trypan blue (Fig. 3A, B, C).

Leaky membranes may also develop at late stages of apoptosis as a result of the global energetic shutdown within dying cells [35]. To further investigate the mechanism of SMFD, we used another dye, toluidine blue, that has been shown to selectively accumulate in apoptotic cells within *Drosophila* tissues [36]. When toluidine blue was applied to cryosectioned thoraces, it developed a uniform staining across muscle tissues and did not preferentially accumulate in degenerative fibers (Fig. 3D, D').

A strong morphological marker of necrosis is mitochondrial swelling [37]. To track mitochondria, we expressed a fluorescent reporter, mito-GFP [25], in all muscles using the *Mef2*-*Gal4* driver. Swollen mitochondria could be detected in degenerative fibers with reduced or lacking F-actin staining (Fig. 3E).

Collectively, our results suggest that SMFD in flies involves compromised sarcolemma and swollen mitochondria and, therefore, is mediated by necrosis.

**Fig. 4:**
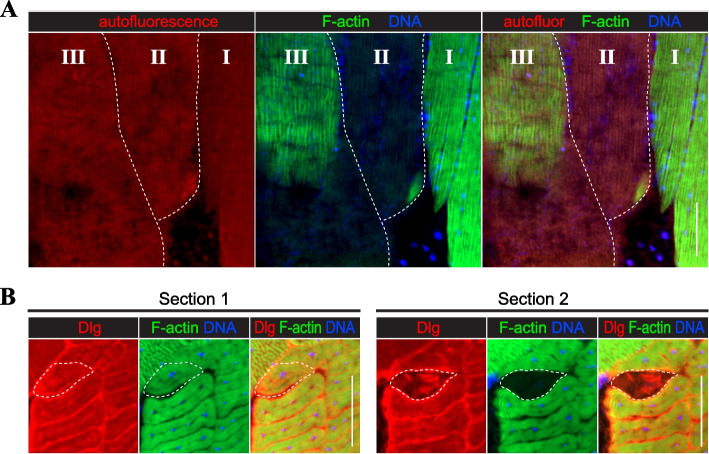
Examples of segmental degeneration of *Drosophila* muscle fibers. **A** Three longitudinally sectioned IFM fibers stained for F-actin (green) and DNA (blue), demonstrating different degrees of degeneration: from no degeneration (fiber I) to complete degeneration (fiber III). Fiber II is partially degenerated, with a segment positive for both F-actin and DNA. Red autofluorescence is used to determine fiber-occupied areas. **B** A fragment of the TDT muscle from two different transverse sections stained for T-tubules (Dlg, red), F-actin (green), and DNA (blue). In section 1, no apparent fiber degeneration is evident; in section 2, a single degenerative fiber is present based on the loss of F-actin and DNA staining and significant perturbations in the Dlg staining pattern. Dashed lines outline degenerative fibers. Scale bars: 50 µm (all panels)

Due to the large size of muscle fibers, injury-induced necrosis can be contained to a segment of a single fiber [38, 39], which is unlikely to occur during programmed death that triggers degenerative changes throughout the entire fiber [40, 41]. Therefore, we analyzed *Drosophila* muscles for instances of segmental degeneration that would serve as the marker of ongoing necrosis. Longitudinally sectioned DLM fibers provided direct evidence of segmental degeneration, demonstrating a dramatic difference in F-actin and DNA staining within the same fiber (Fig. 4A). Using serial sectioning, we could detect cases of segmental degeneration in TDTs as well, in which all markers, including Dlg, remained intact in the unaffected regions of a single TDT fiber but were completely gone or perturbed in the degenerated segment (Fig. 4B). The presence of segmental degeneration further supports the conclusion that SMFD is driven by necrosis.

### Quantitative analysis of SMFD in the TDT muscle

**Fig. 5:**
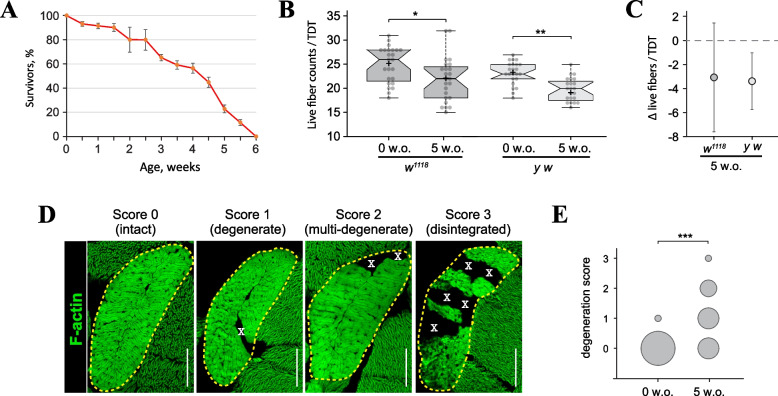
Quantification of spontaneous fiber degeneration in the TDT muscle. **A** Survivorship of *w*^*1118*^ flies reared at 29 °C. Under these conditions, the maximal lifespan is 6 weeks. **B** Box plots of live fiber counts in the TDTs of young (0 week old, wo) and old (5 wo) flies of the *w*^*1118*^ (dark grey) and *y w* (light grey) genetic lines. Crosses depict position of the mean, whiskers are determined by Tukey test, notches show the 95% confidence interval. **C** Data that was re-drawn from B as the average loss of live TDT fibers in old flies. Whiskers show standard deviation. **D** Examples of fiber degeneration in the TDT muscle (outlined by dashed line) with assigned degeneration scores. Degenrative fibers are marked by X.  **E** Quantification of TDT degeneration based on degeneration scores. The area of each circle represents the fraction of muscles received a particular penalty score. Statistical significance by Fisher’s Exact Test: (*) *p* < 0.05; (***) *p* < 0.001. Scale bars: 50 µm

Since adult flies do not have robust regeneration, we hypothesized that degenerated muscle fibers would be especially abundant at the end of the fly’s lifespan. To quantify the extent of degeneration, we chose TDT as this muscle contains the highest number of individual fibers. A common laboratory line *w*^*1118*^ has the maximal lifespan of 6 weeks, when reared at 29 °C (Fig. 5A). In young flies shortly after eclosion (0 wo), the average fiber count per TDT was 25 ± 3.7 fibers, but in old flies (5 wo) the live TDT fiber counts were significantly decreased (22 ± 4.5, *p* = 0.014) (Fig. 5B). A similar decline was observed for *y w* line although it had different absolute fiber counts (23 ± 3.0 vs 20 ± 2.0, p = 2.4 × 10^–5^, Fig. 5B). Direct comparison between different fly lines using live fiber counts is complicated due to such line-specific variability of this parameter. However, when the same data are expressed as the average fiber loss per TDT, the level muscle degeneration become readily comparable across two different fly lines (Fig. 5C).

A more detailed reporting of muscle degeneration can be achieved by scoring TDT muscles based on the number of degenerative fibers and the severity of damage (Fig. 5D). Plotting each damage category on a graph simultaneously demonstrates the quantitative and qualitative differences observed in flies of different ages (Fig. 5E). Notably, this method does not require input from young flies to calculate degeneration rates in old flies and is readily comparable between different lines, significantly streamlining the analysis pipeline (compare Fig. 5E vs C). Therefore, we have selected the penalty scoring method as the method of choice for routine quantitative assessment of muscle degeneration in flies.

### SMFD correlates with chronological and functional aging

**Fig. 6:**
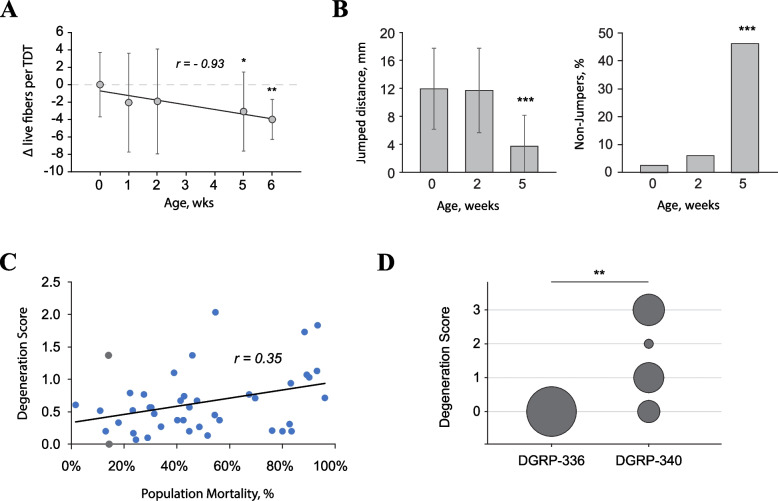
Progressive degeneration of the aging TDT muscle. **A** Changes in live TDT fiber counts within a population of aging *w*^*1118*^ flies. Statistically significant changes are detected in 5 week old (wo) flies. **B** Jump test results obtained from the *w*^*1118*^ flies of different ages. “Non-jumpers” denotes flies that cannot jump. The jumping ability significantly declines in 5 wo flies. Statistical significance by Kruskal–Wallis test (left panel) and Fisher’s exact test (right panel): (***) *p* < 0.001. **C** Scatter plot of TDT degeneration scores *vs* population mortality for 43 DGRP wild-type inbred fly strains reared at 29 °C for 4 weeks. A linear regression line with correlation coefficient (*r*) is shown. Lines that are analyzed in **D** are shown in grey color. **D** Examples of two DGRP lines with comparable survivorship at 4 weeks (13%), but drastically different degeneration scores in the TDT muscle. Statistical significance by Fisher’s Exact Test: (**) *p* < 0.01

Analysis of SMFD occurrences in a population of aging *w*^*1118*^ flies demonstrated a strong correlation between the extent of damage and the chronological age of flies (Pearson’s coefficient = -0.93), although the changes reached statistically significant levels only in old (≥ 5wo) flies (Fig. 6A). A significant advantage of the TDT model is the ability to probe muscle functionality via the jump test, since the TDT (aka “jump muscle”) is the sole muscle executing the escape response in flies [42]. Per jump test results, old flies (5 wo) had a significant decline in the jumping performance and, therefore, TDT functionality (Fig. 6B). These results suggest that although individual fiber degeneration in flies may be well tolerated, eventually the accrued degeneration begins affecting muscle functionality at the gross level, although other attributes of aging, including the neural component, could be at play here.

To probe for a link between SMFD and physiological aging, we used the *Drosophila* Genetic Reference Panel (DGRP), which is a collection of inbred wild-type fly lines with a wide variety of phenotypes, including longevity [43, 44]. We analyzed mortality rates in 43 individual DGRP lines that were aged for a fixed period of 4 weeks at 29 °C. In agreement with previous studies, DGRP lines displayed a range of mortality rates (2–98%), which is indicative of highly variable aging rates among these lines. When the mortality rates were plotted against the SMFD rates obtained from the same flies, a positive correlation (Pearson coefficient = 0.35) was detected (Fig. 6C). These results suggest that SMFD is an aging-related process as more degenerative fibers accumulate in flies with shorter lifespan. Nevertheless, DGRP lines with similar mortality rates may have significantly different SMFD rates, as exemplified with two DGRP lines with similar aging rates (Fig. 6D). This implies the presence of genetic components that influence SMFD rates independently of aging.

### Stimulation of muscle contractile activity increases SMFD rates

**Fig. 7:**
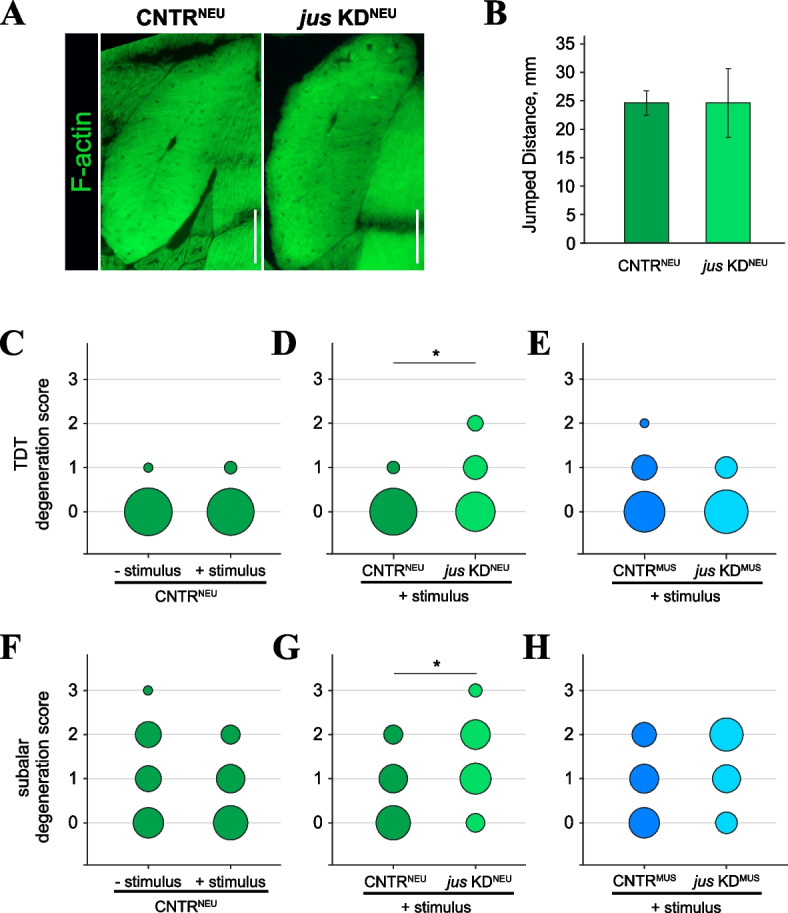
Muscle degeneration is increased in bang-sensitive flies. **A** TDT muscles from flies with *jus* knockdown in the nervous system (*jus* KD^NEU^, *elav* > *jus* KD, pale green) and their isogenic control (CNTR^NEU^, *elav*/ + , dark green) aged for 4 weeks at 29 °C. **B** Jump test results. The *jus* KD^NEU^ flies have fully functional TDTs. **C**, **D** TDT degeneration scores in flies of various genotypes and treatments. “Stimulus” denotes daily vigorous shaking for 10 s to invoke the bang response; *jus* KD^MUS^ is *jus* knockdown in muscles (*Mef2* > *jus* KD, light blue) and their control (CNTR^MUS^, *Mef2*/ + , dark blue), **F**–**H** Quantification of fiber degeneration in subalar muscles from the same flies and under the same conditions as in panels **C**, **D**. Statistical significance by Fisher’s exact test: (*) *p* < *0.05*. Scale bars: 50 µm

The finding that SMFD involves compromised sarcolemma, along with the fact that extensive physical exercise can inflict fiber damage in mammalian muscle [34, 38], suggest that mechanical damage might be the driving force of muscle degeneration in flies. To test this possibility, we used bang-sensitive flies that were prone to seizures upon mechanical stimulation. The bang-sensitive phenotype was induced by tissue-specific knockdown (KD) of the gene *julius seizure* (*jus*) in the nervous system using the pan-neuronal driver *elav*-Gal4 [23]. The TDTs in experimental, bang-sensitive flies (*jus* KD^NEU^) had normal morphology and function (Fig. 7A, B). We aged bang-sensitive flies along with their genetically matched control (CNTR^NEU^) for 4 weeks, while applying mechanical stimulation (brief shaking) daily to stimulate seizures. In control flies, such treatment *per se* did not promote fiber degeneration in the TDT (Fig. 7C), however in bang-sensitive flies we detected a small but significant increase in the levels of TDT fiber degeneration (Fig. 7D).

As a negative control, we used *Mef2*-Gal4 to induce *jus* KD in muscles (*jus* KD^MUS^), reasoning that it should be indifferent since *jus* has no appreciable expression in muscles [45]. Indeed, the *jus* KD^MUS^ flies did not demonstrate bang sensitivity and their TDT degeneration rates were comparable with those of genetically matched control (CNTR^MUS^) (Fig. 7E).

In addition, we quantified fiber degeneration in the subalar muscle. In general, subalar muscles demonstrated a higher basal level of degeneration in comparison to TDTs, but mechanical stimulation in control flies did not further enhance it (Fig. 7F). Similarly to TDTs, in bang-sensitive *jus* KD^NEU^ flies subalar muscle degeneration was significantly higher than in age-matched control (Fig. 7G). Meanwhile, in bang-insensitive *jus* KD^MUS^ flies fiber degeneration in subalar muscles was not different from control flies (Fig. 7H).

Collectively, these data demonstrate that SMFD in flies is elevated by intensified muscle activity. Our results also imply that the functional state of the nervous system is a contributing factor to muscle aging.

### Inhibition of contractile activity does not prevent SFMD

To further explore the contribution of contractile activity toward SMFD, we genetically decoupled the TDT muscle from neurogenic excitation. We did that by knocking down *Ca-α1D*, which codes a subunit of the voltage-gated Ca2 + channel that is critical for muscle contractions [46]. We employed the *Act79B*-Gal4 genetic driver [24] to limit KD effects by the TDT muscle and thus to avoid systemic paralysis and lethality in adult flies.

**Fig. 8:**
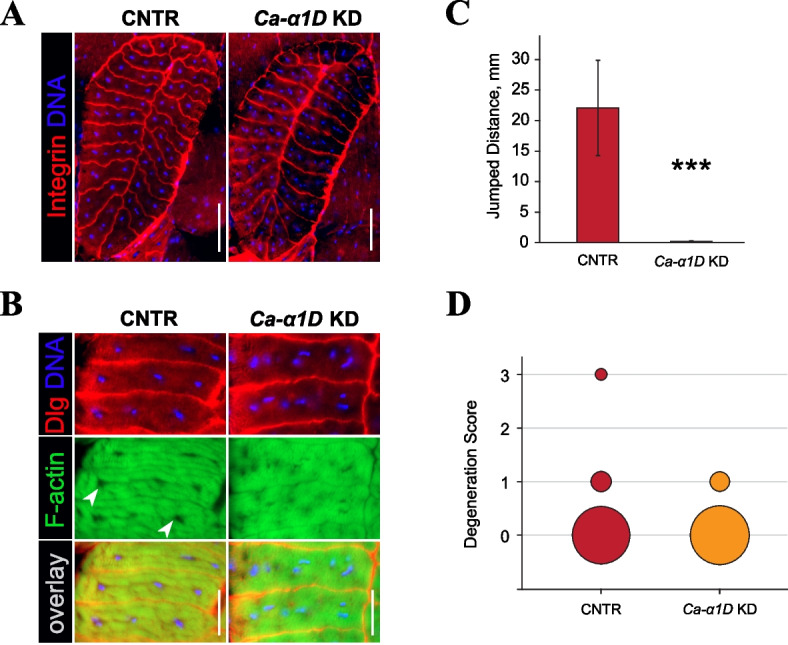
Fiber degeneration persists in non-excitable TDT muscles. **A**, **B** TDT organization in young flies was revealed by F-actin (green), Integrin (red) and nuclear (blue) staining. No changes in muscle cross-sectional area and fiber layout are evident between experimental (*Ca-α1*D KD) and control (CNTR) flies. **B** A closeup of several TDT fibers from young flies stained for F-actin (green), T-tubules (Dlg, red), and nuclei (blue). Organization of myonuclei and myofibrils is perturbed in live TDT fibers of *Ca-α1*D KD flies. Arrowheads indicate nucleus-containing lacunae in the control muscle. **C** Jump test results. *Ca-α1*D KD flies completely lose the ability to jump. Statistical significance by Student’s *t-*test: (***) *p* < 0.001. **D** Degeneration profiles of the TDT muscles in old 4 wo flies. No statistically significant differences between control (CNTR) and experimental (*Ca-α1*D KD) groups were found by Fisher’s exact test. Scale bars: 50 µm (**A**), 20 µm (**B**)

In the *Ca-α1D* KD flies, TDTs developed normally and achieved the usual morphology and size (Fig. 8A). However, the normal distribution of myonuclei in these muscles was perturbed. Instead of 2–3 central lacunae housing myonuclei as seen in control, *Ca-α1D* KD flies had scattered myonuclei that were present at random locations throughout the fiber (Fig. 8B). The viability of fibers containing scattered nuclei was confirmed by positive staining for F-actin and Dlg. Such alteration in myonuclear positioning suggests a disorganization of cellular architecture in the absence of contractions.

TDTs in the *Ca-α1D KD* flies were unable to contract, which was confirmed by the jump test (Fig. 8C). However, aging TDTs in 4-wo *Ca-α1D* KD flies demonstrated SFMD rates similar to those of age-matched genetic control (Fig. 8D).

Since SMFD could not be fully prevented in non-contracting TDTs, it indicates that mechanical stress is not the only factor contributing to fiber degeneration in flies. Some intrinsic muscle factors are apparently at play here as well.

## Discussion

This study characterizes spontaneous degeneration events in the somatic musculature of adult *Drosophila* flies. We reveal that this phenomenon occurs across diverse muscle groups and progresses with age. By using quantitative analysis, we demonstrate that muscle damage correlates with aging (as determined by chronological, functional, and populational criteria) and it is influenced by genetic background and contractile activity. Historically, muscle morphology in *Drosophila* was largely studied using IFMs and in conjunction to muscle development [47–49] or experimentally created degenerative conditions [50, 51]. Our study expands such studies to include tubular muscles TDT and subalar muscles, although we observed signs of degeneration across all somatic muscles, including abdominal muscles (not shown). By comprehensively describing the normal aging-related muscle degeneration, we have laid the foundation for using *Drosophila* in systematic genetic screening to identify factors that affect the quality of muscle aging. We believe that the methodology developed in this study, coupled with the genetic proficiency of the *Drosophila* model, will accelerate discoveries of novel targets for sarcopenia research.

### Markers of muscle degeneration

In this study, we exploited two early markers of muscle degeneration, namely the loss of phalloidin staining of myofibrils and the loss of Dlg immunostaining of T-tubules (we do not consider swollen mitochondria a reliable marker due to the transient nature of the former). Fluorescently labelled phalloidin is a common dye for muscle visualization because it has strong and selective binding affinity toward polymerized actin. Due to a high level of actin sequence conservation, phalloidin successfully stains muscles from virtually all multicellular organisms, including worms, flies, and mammals [52–54]. The phalloidin-binding epitope involves surfaces of three adjacent actin monomers, which makes phalloidin staining sensitive to changes in actin filament organization [55]. Accordingly, phalloidin fails to stain actin filaments in alcohol-fixed cell specimens [56]. Phalloidin staining loss was also reported in response to muscle cell injury in vitro [57] and in vivo [54]. Although we currently do not have a clear mechanistic explanation for phalloidin staining loss in *Drosophila*, it may include 1) full or partial depolymerization of thin filaments in the contractile apparatus, and 2) masking phalloidin-binding epitopes due to confirmational changes in thin filaments. Whether phalloidin staining loss can be a universal marker of muscle fiber degeneration requires further evaluation.

T-tubules are highly dynamic structures that can rapidly change their shape and appearance depending on the conditions [31]. In the early *Drosophila* pupa, larval muscles undergoing programmed remodeling completely lose the T-tubule network within a few hours [58]. Under osmotic shock or after excessive stimulation T-tubules transform into a collection of vacuoles [59–61], which is reminiscent of what we observed in some degenerative fibers (see Fig. 2E), indicating that this is a transient process. One emergent hypothesis explains T-tubule vacuolation as a measure to assist in sarcolemma repair [31]. In our assays, T-tubule network’s disturbance or disappearance was the earliest event in degenerating fibers, and it is likely that T-tubules can serve as an early indicator of muscle injury in other model organisms.

### The mechanism of muscle degeneration in adult flies

Instances of muscle death under normal conditions in *Drosophila* have been previously reported as “programmed muscle death”. Most larval muscles undergo rapid degeneration in response to hormonal cues during metamorphosis in pupae or shortly afterward, in young adults [40, 41, 62]. Under this scenario, fiber degeneration is initiated internally via cell signaling that leads to activation of effector caspases (in apoptosis) or lysosomes (in autophagy) [40, 63]. Similar events take place in anuran larval muscles during tadpole tail degeneration or in mammalian muscle upon denervation or disuse [64, 65].

There is a substantial difference between the programmed death of larval muscles and the degeneration of adult muscles reported in this study. Muscle fibers undergoing programmed death detach, round up, and display extensive fragmentation of their sarcoplasm and nuclei [41]. Notably, the fragmented sarcoplasm remains positive for F-actin staining [40, 41]. Nuclear fragmentation is a universal mark of apoptosis, also reported for dying mammalian muscle fibers [66]. However, none of these morphological changes characterize degeneration of adult muscles described in our study, as the degenerating muscles retain their original shape while completely losing F-actin staining, and their nuclei do not fragment despite reduced DNA content. Additionally, we described cases of segmental degeneration, which is not characteristic of programmed death.

An alternative to programmed cell death is accidental cell death, or necrosis, a form of cell death that is triggered by ruptured outer membranes but not cell signaling [67]. We confirm compromised sarcolemma in degenerative fibers by demonstrating their accumulation of trypan blue. Combined with a lack of selective accumulation of apoptosis-selective toluidine blue, these data strongly suggest that adult muscles die via necrosis. In addition, degenerating fibers contain swollen mitochondria, a morphological hallmark of necrosis [37].

Fiber necrosis, especially segmental necrosis, occurs in skeletal musculature of humans and laboratory animals after extensive exercise [68–70]. Necrotic fibers are also commonly present in human patients with muscular dystrophies and some myopathies [39, 71, 72]. Usually, the presence of necrotic fibers attracts phagocytes [69, 72]. As will be noted below, we did not observe accumulation of cell masses at the sites containing degenerating fibers. This apparent lack of cellular immune response to the presence of dead fibers could be explained by a diminished reactivity of the immune system in adult flies [73].

How do we explain the persistence – albeit at a low level – of fiber degeneration in non-contracting TDTs (see Fig. 8D)? One possibility is that mechanical damage could still be induced in non-contracting TDTs by actively contracting neighboring muscles. The TDT contacts DVMs, subalar muscles (Fig. 1A), as well as several other tubular muscles in the ventral part of the thorax, any of which could transmit mechanical forces to the TDT during their contractions.

Zheng et al*.* [74] reported caspase activation and DNA fragmentation in the tissues of aging *Drosophila* flies, including somatic muscles. Although morphological changes were not analyzed in that study, the authors concluded that apoptotic signaling was active in aging muscles [74]. We do not necessarily see this finding as a contradiction to our own data that favor necrotic muscle fiber death. For example, both apoptotic and necrotic features are found in the degenerating muscles of Duchenne muscle dystrophy [75]. However, a carefully planned study that takes advantage of the *Drosophila* genetics and our quantifiable muscle degeneration model will be necessary to address the role of apoptosis in SMFD.

### No damage repair in Drosophila muscles, bad or good?

Adult somatic muscles in *Drosophila* are traditionally viewed as lacking structural plasticity and regenerative capacity [76]. The latter was recently challenged with a study describing novel adult muscle stem cells in flies [77]. However, during our study, we failed to find signs of muscle repair. Regeneration signatures of injured mammalian muscle involve masses of mononucleated cells concentrating around the site of damage [78]. Indeed, even a small segment of muscle fiber (*e.g.*, TDT) contains hundreds of nuclei and would require an equal number of mononucleated progenitors to repair. However, we did not detect swarms of nuclei around degenerating muscle fibers. We also did not observe intact nuclei *within* degenerative fibers, which rules out a putative repair of muscle fibers by endoreplication, as seen in regenerating cardiac muscle [79]. Therefore, our data support the canonical view, according to which regeneration in adult *Drosophila* muscles is extremely rare. Although this fact separates fly muscles from regeneration-potent vertebrate muscles, it gives a pragmatic advantage to our model: the lack of regenerative capacity enables a lasting record of muscle damage, making the identification of factors affecting structural integrity of muscles more straightforward.

### The effect of mechanical force on muscle architecture

Disconnected and dysfunctional muscle fibers undergo subtle but noticeable changes in their morphology. In mammals and humans, denervated muscle fibers reduce in size and assume atypical, angular shapes in muscle cross-sections. Upon long-term denervation, fibers with centrally located myonuclei appear [80]. In *Drosophila*, the physical connection to the nervous system is crucial during early myogenesis for determining the type and final size of developing muscle [81]. However, much less is known about the requirement of neurogenic activation for the maintenance of fully formed adult *Drosophila* muscles, given their notoriously low structural plasticity [24]. In this study, we demonstrate that disabling neurogenic contractions affects nuclear positioning, resulting in scattered myonuclei in the TDT muscle.

In mammals, myonuclei move between peripheral and central regions of the muscle fiber during development and regeneration [82]. Nuclear movement is important for normal muscle functioning since its misregulation leads to centronuclear myopathies in humans [83, 84]. *Drosophila* was instrumental in dissecting the genetic control of myonuclear positioning by identifying the key factors participating in this process [85, 86]. However, the gene *Ca-α1D* that was targeted by RNAi in our experiments was not previously implicated in myonuclear positioning. Therefore, it might be that the nuclear misalignment observed in the dysfunctional TDTs results from non-genetic factors. Indeed, mechanical forces have been shown to determine myonuclear positioning within mammalian muscle fibers [87]. It is intriguing to hypothesize that contractile forces determine the nuclear localization within TDT fibers as well, despite some architectural differences displayed by *Drosophila* [24] and mammalian [88] muscle fibers.

### The role of the nervous system in muscle aging

Our observations imply that mechanical damage stimulates muscle degeneration in aging flies. The nervous system regulates the intensity and duration of muscle contractions and may modulate the amount of mechanical stress received by muscles. Using bang-sensitive, seizure-prone flies, we could demonstrate how a compromised nervous system can affect and promote muscle degeneration. A coincidental muscle damage from seizures was also reported in humans [89]. Although seizures are the extreme means to inflict muscle damage, we hypothesize that even subtle deviations from the normal neurogenic activation of muscles could influence the wear-and-tear of muscle fibers, if continuously occurred over the lifetime.

How relevant is the link between the nervous system and fiber degeneration in the context of mammalian muscle? Upon acute injury, mammalian muscle can effectively repair damaged fibers and replace dead fibers [10]. However, chronic or recurring damage can eventually overwhelm the regenerative capacity, as seen in the case of Duchenne and other progressive muscle dystrophies [12]. Furthermore, the efficacy of muscle regeneration may decline with age [11], potentially leading to the reduction of fiber counts reported for older adults [13].

Currently, genetic factors affecting the normal functioning of the nervous system are not considered *bona fide* candidates for sarcopenia [90], although neurogenic etiology of sarcopenia has been proposed [91, 92]. Embracing the nervous system as a driver for muscle degeneration may substantially expand the range of potential candidates for sarcopenia.

## Conclusions

Based on our results, *Drosophila* muscles experience damage throughout the fly’s lifetime, resulting in stochastic degeneration and death of individual muscle fibers. Mechanical stress from muscle contractions contributes to such degeneration, but the extent of this effect is largely modulated by genotype. The model and approaches described in our study can be utilized for identifying genes that influence muscle resistance to mechanical damage and enhance muscle functional longevity.

## Data Availability

No datasets were generated or analysed during the current study.

## Abbreviations

BDSC: Bloomington *Drosophila* Stock Center
BSA: Bovine Serum Albumin
CCD: Charge-coupled device
CNTR: Control
DAPI: 4',6-Diamidino-2-phenylindole
DGRP: *Drosophila* Genetic Reference Panel
DLM: Dorsal Longitudinal Muscle
DVM: Dorso-Ventral Muscle
IFM: Indirect Flight Muscle
KD: (Genetic) knockdown
OCT: Optimal Cutting Temperature
PBS: Phosphate Buffered Saline
RNAi: RNA interference
SDH: Succinyl dehydrogenase
SMFD: Spontaneous muscle fiber death
TDT: Tergal depressor of the trochanter
wo: Week(s) old
